# Genomic Evolution and Epidemiological Impact of Ongoing Clade Ib MPox Disease: A Narrative Review

**DOI:** 10.1155/ghe3/8845911

**Published:** 2025-05-15

**Authors:** Adewunmi Akingbola, Adegbesan Abiodun, Courage Idahor, Favour Peters, Olajide Ojo, Otumara Urowoli Jessica, Uthman Hassan Alao, Olajumoke Adewole, Abdullahi Owolabi, Joel Chuku

**Affiliations:** ^1^Department of Community Health, University of Cambridge, Old Schools, Trinity Lane, Cambridgeshire, Cambridge CB2 1TN, UK; ^2^African Cancer Institute, Department of Global Health, Stellenbosch University, Cape Town, South Africa; ^3^Nottingham University Hospitals NHS Trust, Nottingham, UK; ^4^University of Cambridge, Old Schools, Trinity Lane, Cambridgeshire, Cambridge CB2 1TN, UK; ^5^University of West England, Coldharbour Ln, Stoke Gifford, Bristol, UK; ^6^Faculty Of Allied Health and Social Care, Anglia Ruskin University, Cambridge CB1 1PT, UK; ^7^Department of Biomedical Laboratory Science, University of Ibadan, Ibadan, Oyo, Nigeria; ^8^Department of Community Health, Lagos State University College of Medicine, Ikeja, Lagos, Nigeria; ^9^Faculty of Clinical Sciences, Bayero University, Kano, Nigeria; ^10^Department of Medicine, V. N. Karazin Kharkiv National University, Svobody Square, Kharkiv 61022, Ukraine

**Keywords:** clade 1b MPox, global outbreak, public health emergency, surveillance, vaccine access, virus transmission

## Abstract

Clade 1b of the MPox virus has emerged as a highly virulent strain, causing significant public health challenges globally. Initially endemic to Central Africa, this strain has spread to nonendemic regions, including Europe, Asia, and the Americas. With its high transmission rate and severe outcomes, especially among vulnerable populations like children, Clade 1b has raised global concerns. The Africa Center for Disease Control and Prevention (CDC) has declared it a public health emergency of international concern. Clade 1b MPox shows a higher case fatality rate and increased transmissibility compared to other strains. It has moved beyond traditional zoonotic transmission to widespread human-to-human transmission. The variant's spread to countries such as Sweden and Thailand demonstrates its global reach. Public health efforts, including cross-border coordination, rapid response teams, and awareness campaigns, have been essential in containing the outbreaks. However, barriers such as limited resources, vaccine shortages, and logistical challenges in conflict-affected areas have hindered effective control, particularly in low-resource regions. The spread and severity of Clade 1b MPox highlight the need for global cooperation to strengthen surveillance, improve diagnostic capabilities, and expand healthcare infrastructure in affected areas. Enhancing access to vaccines and treatments, along with educating the public on preventive measures, will be key to controlling transmission. Ongoing research and monitoring are essential to mitigate future outbreaks and minimize the virus's global impact.

## 1. Introduction

**Figure 1:**
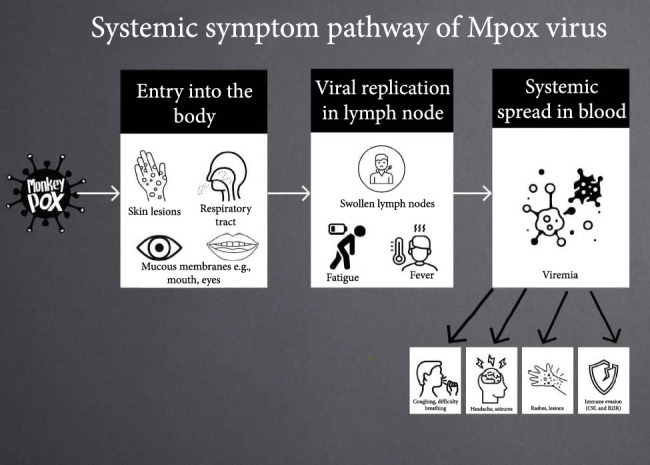
Illustrates the progression of MPox infection from entry into the body, replication in lymph nodes, and systemic blood spread, leading to severe symptoms and immune evasion mechanisms.

The monkeypox (MPox) virus is a frequently overlooked zoonotic virus that leads to a disease resembling smallpox in humans, albeit with less severity. Initially identified in 1958, MPox is a double-stranded DNA virus belonging to the Poxviridae family, the chordopoxvirinae subfamily, the Orthopoxvirus genus, and the MPox species [1]. The first recorded case of MPox in humans was noted in the Democratic Republic of Congo (DRC) in 1970 [2], with later outbreaks confined mostly to Africa, particularly among children in rural regions [1]. Since its discovery, various transmission methods have been recognized, evolving from early close contact with infected individuals, animals, or carcasses to recent sexual transmission, predominantly among gay, bisexual, and men who have sex with men (GBMSM), as well as commercial sex workers [2, 3]. While the definitive host for MPox has yet to be pinpointed, several wild reservoirs have been suggested, particularly rodents, rendering MPox a sylvatic disease [3].  Figure 1 below shows the transmission pattern and how it causes the symptoms.

Two primary variants of MPox have been identified: Clade I and Clade II. Clade I is endemic to the DRC and neighboring countries such as the Republic of Congo, Central African Republic, and Gabon [4]. Clade II, which includes subclades IIa and IIb, is predominant in Western African nations like Nigeria, Cameroon, Ghana, and Côte d'Ivoire [4]. Clade I hold significant importance due to its elevated potential for transmission and high mortality rates. Clade I have an estimated case fatality rate (CFR) of 10% in unvaccinated individuals, and alongside confirmed human-to-human transmission [4]. Moreover, outbreaks of the Clade I MPox virus are increasing in Central and East Africa, thereby maintaining a risk of cross-border and international spread [5]. Worldwide, MPox has been characterized primarily as a disease endemic to the forested regions of Central and West Africa [3]. However, in recent periods, cases have been reported across various continents. In May 2022, there was a significant outbreak of MPox that impacted over 70 countries around the globe. This prompted the World Health Organization (WHO) to declare the disease a public health emergency of international concern (PHEIC) on July 23, 2022 [3]. As of April 30, 2024, the disease has affected over 97,745 individuals across borders into American, European, and Asian nations [2].

This review aims to explore the phylogenetic traits and epidemiological consequences of Clade I. Gaining insights into the epidemiology, genetic evolution, and diversity of this clade is vital for the creation of targeted interventions, improving diagnostic precision, and customizing public health strategies to mitigate further transmission and reduce the disease's impact in both endemic and nonendemic regions.  Table 1 below summarizes the key insights, the methodologies, and main findings in a number of studies which have formed a foundation basis for writing this paper.

## 2. Genomic Overview of the MPox Virus

### 2.1. Phylogenetic Description of MPox Virus

**Figure 2:**
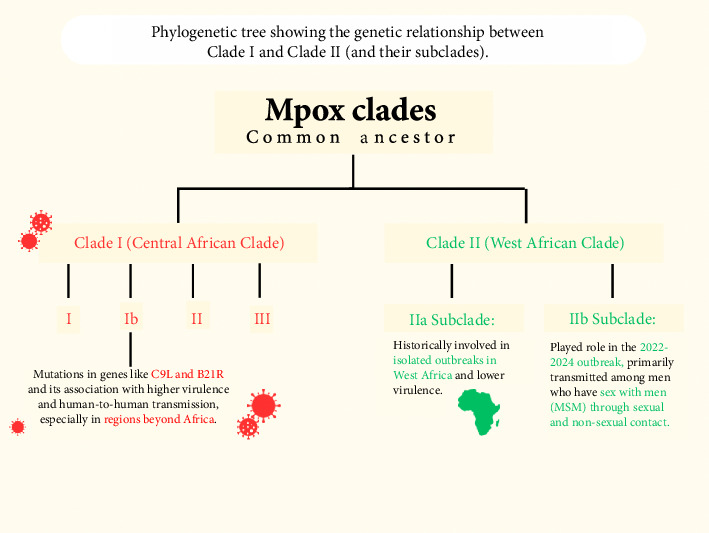
A visual representation of the genetic relationship between Clade I (Central African Clade) and Clade II (West African Clade), highlighting key mutations and transmission patterns.

The monkeypox virus (MPXV) consists of a large, linear double-stranded DNA genome that falls under the genus orthopoxvirus (OPV), family poxviridae, and subfamily chordopoxvirinae [6]. This viral genome is about 200 kilobases long and contains approximately 200 genes, translating to the potential for encoding around 150-190 proteins [7]. It has around 190 open reading frames (ORFs) critical for functions such as replication, morphogenesis, immunomodulation, and pathogenesis [8]. There are two distinct classified clades, or genetic variants, of MPXV that are indigenous to Africa: Clade I (previously referred to as the Congo Basin or Central Africa Clade) and Clade II (West African Clade). Clade I MPXV is further categorized into six subgroups, designated I, II, III, IV, V, and more recently, VI, which emerged following an observational cohort study in Kamituga, DRC [6, 9]. Clade II is also divided into two subclades: IIa and IIb [10]. These MPXV clades display different geographic, epidemiologic, and clinical characteristics. The Clade IIa variant was responsible for localized outbreaks in Nigeria, Ivory Coast, Sierra Leone, Liberia, and the United States [10]. However, due to the MPXV outbreak occurring in 2022–2024, a new subclade, IIb, was identified as part of the genetic variants involved [11]. The Subclade IIb MPXV was predominantly identified in men who have sex with men (MSM) and among individuals who engage in sexual contact with infectious persons [12]. As a result, Clade IIb is primarily transmitted between humans through both sexual and nonsexual interactions with an infected person, whereas Clade I and IIa MPXV tend to spread less among humans and are mainly transmitted zoonotically through rodents and small mammals [1]. Clade I MPXV is linked to more severe disease presentations and has an elevated CFR averaging 10.6%, compared to Clade IIb, which has a lower CFR of approximately 3.6% [13]. Clade I is endemic to the DRC, Central African Republic, Republic of Congo, Sudan, Cameroon, and Gabon and possesses smallpox-like attributes with a high occurrence of viremia [10]. Clade IIa presents milder symptoms, reduced viremia and transmissibility, and a much lower CFR (around 1%–3%) in comparison with Clade I MPXV [14, 15].  Figure 2 below shows the relationship between Clade I and Clade II.

### 2.2. Genetic Features of Clade 1 MPox

Clade 1 of the MPox virus exhibits a distinctive genetic composition that sets it apart from other clades. In the ongoing MPox outbreak, clusters of transmission through heterosexual contact (which was not a common observation) have been recorded and linked to a novel sublineage termed Clade Ib, which has spread to countries like Sweden where MPox cases were not previously recorded [16]. Clinical reports have described Clade Ib symptoms as disseminated whole-body rash, long-lasting genital lesions and outcomes including pregnancy loss in pregnant women, with a CFR of up to 10% in children and 5% in adults [17]. Given these new disease outcomes, demographic patterns, modes of transmission, and international spread, it is imperative to elucidate the genetic factors in Clade I contributing to this increased pathogenicity.

Clade I contain several key structural and functional genes that play crucial roles in its virulence and pathogenicity. Notable among these is the C9L gene, the only Kelch-like protein in the MPXV genome that acts as an inhibitor of the host innate immune response [18, 19]. The I4L gene, which encodes a ribonucleotide reductase (RR) large subunit 1 protein, is involved in viral replication in nondividing cells [20]. Although further studies are needed to investigate the specific contribution of the A25R gene to MPXV virulence and pathogenicity, it is believed to be fundamental to MPXV's ability to replicate its DNA into RNA [21]. The central location of A25R in the core region of the viral genome and its high conservation across various MPox strains highlights its critical role in the viral life cycle and, thus, can be an important potential target for vaccine development and drug therapy [22]. Similarly, the role of the L6R gene is not fully understood; however, it is predicted to be essential for facilitating viral DNA replication and transcription [23]. The presence of the A17L (a viral envelope protein) in vaccinia virus (VAVC), which is involved in host cell entry and fusion, plays a similar and essential role in MPXV in determining intracellular sites for cleavage, disulfide bond formation, and infection onset [24]. A recent study conducted by de Araujo et al. [25] identified numerous epitopes from the A28L protein and proposed them as candidates for a multi-epitope vaccine, highlighting their potential for immunogenicity. In silico analyses have also revealed that these epitopes could trigger both humoral and cellular immune responses, making them a promising component in the development of an immunogenic construct against MPXV. This follows that the genetic diversity in MPXV Clade I is integral to viral transmission and survival within the host as well as its ability to suppress immune responses, infect host cells, and replicate, underscoring the significance of these genes in the overall genomic architecture of MPXV Clade I.

### 2.3. Genetic Variability and Significant Mutations Differentiating Clade I

As the most virulent clade of the MPox virus, Clade I have been associated with more severe disease and transmission outcomes. Genetic variability is one of the prominent factors that influence the epidemiological behavior of the ongoing MPox Clade Ib outbreak. Notably, this Clade Ib outbreak has been self-sustaining, exhibiting unusual characteristics rarely observed in previous Clade I outbreaks (which were primarily due to zoonotic transmission), such as human-to-human transmission through heterosexual contact, nonsexual community spread to geographic areas not previously reported such as in Europe, Asia, and the Americas, and infecting over 99, 176 people, across varying age groups including children [26, 27]. This paper describes the specific mutations or genetic markers in MPXV Clade 1 that makes the epidemiological profile of this new sublineage outbreak distinct from other previously observed Clade I outbreaks. A mutational analysis reported by Masirika et al. [6] revealed that MPXV Clade I (most importantly Clade Ib) contain a set of highly mutative proteins including C9L [OPG047], I4L [OPG080], L6R [OPG105], A17L [OPG143], A25R [OPG151], A28L [OPG153], and B21R [OPG210]). In comparison with the MPXV Clade I genome (NC_ 003310), these mutational hotspots contain a number of consensus inframe deletions, frameshift variants, synonymous variants, and amino acid substitutions. The B21R (OPG210) protein, which codes a surface glycoprotein, was found to be associated with the highest number of mutations including two consensus frameshift variants (K431X and P726X), synonymous variants (A1207 and C1715), and nine amino acid substitutions (A475V, S533L, T713P, T723P, E1056D, A1339T, T1372A, V1725S, and D1862E).

When compared with the reference genome, B21R (OPG210) contains a total of four unique changes, two frameshift variants (K431X and A475V), and two amino acid substitutions (P726X and E1056D). C9L (OPGO47), which is a Kelch-like protein, was found to have an inframe deletion (DGMG483-486E) and one frameshift variant (D483X) with three consensus amino acid substitutions (L151H, V127E, and Y114N). This protein, in comparison with subgroup V (JX878417), was found to have a unique inframe deletion [27]. Fourteen rare genes (D2L, D4L, D17L, D18L, C3L, A26L, A27L, A48R, B15L, B1R, B18R, K1R, R1R, and N2R) not previously observed for Clade I MPXV were also present in the genome samples and were found to be unique to the ongoing MPox Clade Ib outbreak. These mutational variabilities are reported to be likely responsible for the sustained nature, unusual transmission routes, and high virulence of the current Clade 1 outbreak [28]. Recently, Zhang et al. [25] conducted a phylogenetic analysis incorporating 84 locally sequenced MPXV Clade I genomes from Beijing. One of these (identified as an imported infection) exhibited 76 nucleotide substitutions and 34 amino acid substitutions relative to the reference genome (NC_063383.1) and included changes such as C33332T (OPG050:R26Q), G37974A (OPG056:S354L), and C149963T (OPG0176:S52L), which were not present in the previous Beijing sequences. Other studies have shown that Clade I exhibit a moderate level of genetic diversity, with variability primarily concentrated in the B21R and A25L gene regions [27, 29]. This variability is attributed to a combination of point mutations and insertion–deletion events that have occurred over time [8, 30]. It is reasonable to suggest that specific mutations, such as those in the C9L and B21R genes, can be linked to changes in viral protein expression and function, which may impact Clade Ib virus's ability to evade host immune responses and exhibit more severe disease outcomes.

### 2.4. Comparative Context Between the Clade I and Clade II

Several studies have highlighted the genomic differences between Clade I and Clade II MPXV (including subclades IIa and IIb) and the potential correlation to virulence differences. Nine proteins that varied by at least five amino acids between the two clades were reported by Likos et al. [14]. Based on orthologs in the VAVC, four of them (OPG110, OPG135, OPG180, and OPG164) were reported to play functional roles in the viral life cycle and five were predicted to be involved in immune evasion or host range, including complement activation and apoptosis regulation. Other investigations have found OPG195, B14R, and OPG032 (which vary between clades I and II) to be determinants of MPXV virulence. Chen et al. [21] discovered that virulence differences between Clade I and Clade II MPXV likely originated from genes found in the terminal regions of the viral genome. Of these genes, two are fragmented in Clade IIa MPXV (OPG195 and B14R) and one (D14L) is absent. D14L has been identified as a likely candidate for virulence differences as this gene is present in Clade I MPXV but absent in Clade II MPXV. Additional sequence differences have been reported in nonvirulence orthologs related to the viral life cycle between Clade I and recent Clade IIb viral genome sequences. In terms of functional roles of MPXV virulence determinants, B10R, which is believed to abrogate apoptosis in infected cells based on homology to the myxoma virus M-T4 protein, encodes a 221 amino acid (aa) protein in Clade I viruses but is fragmented in Clade IIb viruses [21]. This follows that MPXV virulence factors may have heterogeneous roles in clade-specific contexts. Thus, functional characterization of the roles of putative virulence proteins in Clade IIb MPXV needs to be further studied. While other clades exhibit higher genetic diversity in certain genomic regions, Clade I maintain a relatively stable genome structure, suggesting a different evolutionary trajectory compared to Clade II, which has shown some recombination events [31]. These comparative insights highlight the evolutionary distinctiveness of Clade I and its implications for epidemiological monitoring and control strategies. The degree of genetic diversity within Clade I reflects its adaptive evolution and provides insights into its transmission dynamics and potential for outbreak resurgence. Identifying these mutations can enhance the understanding of how Clade I interact with the host immune system, evades immune responses, and adapts to different environmental pressures. This is not only vital for more accurate epidemiological surveillance but also for informing vaccine development, antiviral therapies, and diagnostic tools.  Table 2 provides an overview of the key differences between Clade I and Clade II MPXV.

### 2.5. Drivers of Evolution; Recombination, Gene Loss, and Gene Expression

Recent advances in genomic sequencing have provided critical insights into the evolutionary dynamics of MPox Clade I, enhancing knowledge of MPXV genetic diversity and adaptation. As a highly virulent clade, exhibiting more severe clinical manifestations, Clade I have been the focus of extensive research, particularly in regions where it is endemic. The application of high-throughput sequencing technologies has allowed for easy tracking of mutations, identification of genetic markers, and understanding of the evolutionary pressures shaping Clade I MPXV. Double-stranded DNA viruses such as MPXVs exhibit a slow rate of evolution. However, to adapt to human hosts, MPXVs undergo microevolution, involving amino acid point mutations [34]. To quickly gain the first insights into phylogenetic placement and evolutionary tendencies of the MPXV, Yu et al. [3] classified frequent amino acid mutations into four groups based on their dates of first appearance which were determined through evolutionary analysis. Evidently, the incidence of amino acid mutations conserved in the MPXV genomes over time increased significantly between 2021 and 2023. The occurrence of such multiple genetic changes within this short period, which is not frequently observed in OPVs or particularly in MPXVs, implies that the MPox virus has acquired enhanced rapid host adaptation and gained a fitness advantage that facilitates its ability to maintain human-to-human transmission [8,30]. This rapid sequence of alterations is probable to have played a critical role in MPXV host adaptation, spread in dynamic and changing environmental conditions, and consequently, the higher transmission and infection establishment of Clade Ib in the human population. Since 2017, MPXV Clade IIb genomes have been observed to consistently accumulate apolipoprotein B editing complex (APOBEC3)-type mutations, induced by host defense mechanisms. These molecules target the viral genome during replication when single strands are exposed. Either of these strands can undergo deamination, which progressively causes changes, such as cysteine conversion to thymine or guanine to adenine [34]. Although APOBEC3-mutated genomes have been reported to be nonviable, these genomes may persist and get transmitted. Constant evolution within the human population, due to the irreversibility of APOBEC3, can result in a potential decline in the virus fitness. The timeline for this process remains an area for further studies and additional evolutionary factors such as recombination could inhibit this decline and restore virus fitness [32]. Further research into the mechanisms governing genome evolution and the significance of gene functions is also crucial to gain a deeper understanding of the processes involved in MPXV evolution.

### 2.6. Recombination

Recombination is predicted to be one of the main drivers of poxvirus evolution [35]. Although poxviruses undergo high-frequency recombination during cell infection, no natural recombination events have been reported for MPXVs and little is known about the exact role of recombination in MPXV evolution [36]. Nevertheless, few studies have attempted to engineer recombinant MPXVs in the lab such as Goff et al. [29] who replaced the D14L gene with an EGFP-GPT cassette in the MPXV Clade I genome using homologous recombination to examine the involvement of the MOPICE (monkeypox inhibitor of complement enzymes) in MPXV pathogenesis. This generated a recombinant MPXV-producing green fluorescent protein to analyze MPXV infection in a monkey model. The ease of constructing recombinant MPXVs raises the possibility that such recombination can occur or may have occurred naturally between co-infecting MPXVs. Yeh et al. [33] reported the first natural recombination of the MPXV genome using SNP-dependent and SNP-independent analysis tools, including linkage disequilibrium (LD) and tandem repeat (TR) analysis, speculating that the progeny MPXV recombinants emerged from a single origin which gained mutations, evolved into different lineages, and then went through homologous recombination through multiple possible mechanisms. The resulting recombinants, however, had mosaic patterns of TRs and no defective MPXV virus was detected arising from a single infection.

### 2.7. Gene Loss and Gene Expansion

Another driver of poxvirus evolution is believed to be gene loss, and gene gain as is the main form of host adaptation [37]. Gene content and genome sequence length have an inverse relationship with pathogenicity [38]. MPXV Clade II have larger genomes, approximately between 197,566 and 197,792 bp, more than Clade I (∼196,850–196,959 bp) which contributes to the high virulence of Clade I MPXV when compared to Clade II [36]. It is not unreasonable to assume that poxviruses (MPXV in particular) adopt reductive evolution by gene loss to enhance their response to, and evade, host immune response mechanisms. Hatcher et al. [39] provide strong evidence that gene loss primarily occurs through the introduction of ESMs (early stop mutations) leading to truncations, fragmentations, and complete deletion of protein-coding regions (ORF). A study demonstrated that under intense selective pressure, poxviruses can implement temporary gene expansion to counteract host antiviral response [40]. For example, deletion of E3L in VAVC was observed to increase the copy number of K3L, expanding its genome size by up to 10% which inhibited the antiviral defenses of PKR (human protein kinase R). It is plausible that this gene expansion mechanism may occur in MPXV and be a driver of evolutionary adaptation. Next-generation genome sequencing and mutational analysis studies are vital for monitoring the emergence of new variants, predicting potential outbreaks, and to better understand the mechanisms driving pathogenicity and response to environmental changes, ultimately contributing to more effective public health strategies, and developing targeted interventions.

## 3. Epidemiological Implications

MPXV is traditionally endemic to Central Africa, especially the DRC, which along with multiple neighboring countries has been suffering an outbreak of MPXV Clade II since 2022 [41]. However, in September 2023, an increase in cases was found to be caused by a new offshoot of MPXV Clade 1, called Clade 1b [42]. Following the continued spread of the disease and spread to neighboring countries, the Africa Center for Disease Control and Prevention (CDC) recently declared MPox a PHEIC [43]. This new variant is of particular concern because it appears to be both more severe and easily transmissible compared to other clades of MPox. MPXV Clade 1b has a higher CFR of up to 10.6% compared to that of Clade II which is 1% [41, 44, 45]. As of 23rd August 2024, Africa CDC reported a total of 20,720 cases across 13 member states, which resulted in 582 deaths; this is a CFR of 2.81%. The vast majority of these cases have occurred in DRC, which has 19667 cases and 575 deaths, that is 95% of the total cases and 99% of the deaths [46]. The higher transmissibility of the Clade 1b variant is evidenced by the fact that the first 6 months of 2024 saw a greater number of cases for the new variant than the total number of cases seen in 2023 [42]. The Clade 1b variant has also disproportionately affected children compared to its counterpart, it is reported that 67% of suspected cases, and 78% of all MPXV Clade 1 suspected deaths have occurred in individuals 15 years old or younger [47].

**Figure 3:**
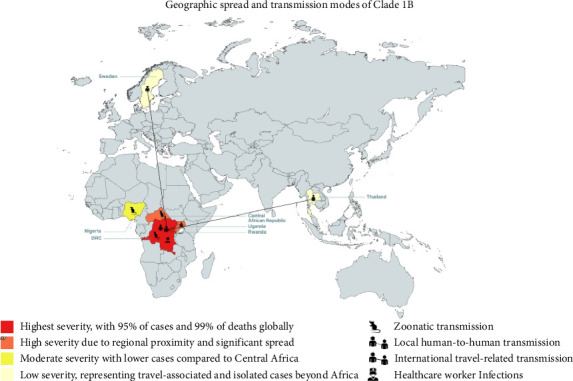
Global spread and transmission patterns of Clade 1B: This map illustrates the geographic distribution and severity of Clade 1B infections.

Clade 1 MPXV is also of international concern because it has already spread to countries immediately neighboring the DRC, such as the Central African Republic, Rwanda, and Uganda and even countries further afield like Nigeria, as shown in Figure 3 below. Beyond Africa, in the second week of august, both Sweden and Thailand also reported confirmed cases of MPXV Clade 1 within their borders in individuals who had recently traveled to African countries with confirmed cases of the disease [48]. Transmission of the disease may occur via a number of routes, they are primary zoonotic transmission, sexual contact with an infected person, and nonsexual human-to-human contact, and there are documented cases of healthcare workers contracting the virus via care of diseased patients [46]. The Africa CDC, WHO, and affected countries have been able to mount rapid and capable responses to the new outbreak. Common approaches include public health emergency operations and rapid response teams, public information campaigns, and cross-country coordination [44]. Unfortunately, a lack of funds and resource scarcity remains a limitation in certain countries. However, WHO and Africa CDC support is aimed at assisting struggling countries. The Africa CDC recently approved 10.4 million dollars to support ongoing efforts to combat the outbreak, and WHO has been instrumental in facilitating labs with necessary reagents and equipment [44, 46]. Finally, vaccine access unfortunately remains a significant challenge due to limited global availability, and the logistical challenges involved with the often remote and conflict-affected areas affected by the disease spread [48].

## 4. Recommendations

Given the swift progression and heightened virulence of Clade 1b MPox, it is essential to emphasize genomic surveillance as a fundamental approach for tracking its dissemination and mutation trends. Ongoing genomic sequencing, especially in areas where the virus has recently appeared, will aid in detecting mutations and forecasting potential outbreaks. Governments and public health entities should allocate resources to enhance access to genomic tools and foster collaboration among laboratories worldwide to monitor new variants in real time. This will empower health authorities to react more swiftly and customize public health strategies based on the specific genetic characteristics of Clade 1b. Furthermore, strengthened public health initiatives are vital to limit the transmission of Clade 1b, particularly in light of its new human-to-human transmission pathways. Awareness campaigns should be launched to inform communities about the dangers of direct contact, sexual transmission, and other unconventional methods of virus spread. Moreover, efforts should be directed at enhancing diagnostic capabilities in both endemic and nonendemic areas, ensuring that healthcare professionals can promptly identify and isolate cases of Clade 1b to avoid further spread. Special focus should be placed on at-risk groups, including children and healthcare workers, who have faced a greater impact in recent outbreaks. Lastly, there is an immediate necessity for research and innovation in the development of targeted vaccines and treatments specifically aimed at addressing Clade 1b. The mutations identified in critical genes such as C9L and B21R indicate that existing vaccines may not be as effective against this changing variant. Scientists should concentrate on creating multi-epitope vaccines and antiviral treatments that can respond to the distinct genetic profile of Clade 1b. Governments and international entities must also ensure equitable distribution of these vaccines and treatments, particularly in resource-limited regions where the virus is most widespread, to reduce global transmission and lower mortality rates.

## 5. Future Perspectives

As the global epidemiology of Clade 1b MPox continues to evolve, future research must focus on understanding the genetic drivers of its increased transmissibility and virulence. Advanced genomic sequencing and phylogenetic studies will be critical in identifying novel mutations that enhance the virus's ability to evade immune responses. Additionally, the role of environmental and ecological factors—such as climate change, urbanization, and human–wildlife interactions—should be explored to determine their contribution to the virus's spread. Strengthening real-time genomic surveillance systems will allow for early detection of emerging variants, facilitating proactive public health interventions rather than reactive containment measures. From a clinical and public health standpoint, the development of targeted vaccines and antiviral therapies for Clade 1b MPox is urgently needed. Existing vaccines designed for Clade II MPox may not provide sufficient protection against this more virulent strain, necessitating further immunogenicity and efficacy trials. Moreover, increasing global collaboration and equitable vaccine distribution will be essential in mitigating future outbreaks, particularly in resource-limited settings where Clade 1b is most prevalent. Research into long-term immunity and potential reinfection risks will also be crucial in shaping future prevention strategies. Ultimately, a multidisciplinary approach, integrating virology, epidemiology, public health, and evolutionary biology, will be key to understanding and controlling the continued threat posed by Clade 1b MPox.

## 6. Conclusion

This study highlights the genetic evolution and increasing severity of the Clade 1b MPox virus, marking a concerning shift in its transmission and pathogenicity. The emergence of Clade 1b, characterized by significant mutations such as those found in the C9L and B21R genes, has allowed the virus to adapt more effectively to human hosts, enhancing its ability to evade immune responses. These mutations, alongside frameshift variants and amino acid substitutions, have contributed to the increased virulence of Clade 1b, resulting in more severe disease outcomes, such as higher mortality rates and long-lasting symptoms. Unlike previous outbreaks primarily driven by zoonotic transmission, Clade 1b has demonstrated a greater capacity for sustained human-to-human transmission, including through heterosexual contact—an unusual feature not commonly associated with earlier Clade 1 outbreaks.

The study also indicates that the evolution of Clade 1b is driven by several genetic factors, including environmental pressures and viral adaptations to host immune defenses. The rapid accumulation of mutations, particularly in key genes responsible for immune evasion and viral replication, suggests that the virus is undergoing microevolution to maintain its fitness in human populations. This enhanced adaptability, combined with new modes of transmission, has contributed to the virus's spread to regions beyond its original endemic areas. Continued research is essential to further explore the evolutionary mechanisms behind Clade 1b′s development and to inform targeted interventions aimed at preventing the spread and severity of future outbreaks [49].

## Figures and Tables

**Table 1:** Summary of key studies on Clade 1b MPox.

Characteristic	Clade I	Clade II
Geographic distribution	Endemic in Central Africa (DRC, Gabon, CAR, Sudan)	Endemic in West Africa (Nigeria, Ghana, Cameroon)
Virulence	Higher virulence and mortality	Lower virulence and mortality
Case fatality rate (CFR)	∼10.6% (higher in children)	∼1–6%
Transmission mode	Traditionally zoonotic; increasing human-to-human transmission	Primarily human-to-human (including sexual transmission)
Genomic features	More conserved genome; fewer mutations	Greater genetic diversity; some recombination events observed
Immune evasion mechanisms	Stronger immune evasion genes (e.g., C9L, B21R)	Weaker immune evasion mechanisms
Clinical presentation	Severe disease; higher rates of virulence, systemic symptoms, and pregnancy complications	Milder disease; localized lesions, lower levels of virulence
Spread beyond Africa	Rare before recent outbreaks (Clade 1b now spreading)	Widespread global outbreak since 2022
Vaccine effectiveness	Current vaccines may have reduced effectiveness due to immune evasion genes	More responsive to existing smallpox vaccines
Epidemiological concerns	Emerging variants (Clade 1b) with unusual human transmission patterns	Ongoing but lower mortality compared to Clade I

**Table 2:** Comparison of Clade I and clade II MPox virus.

Study	Year published	Methodology	Main findings	Relevance to topic
Likos et al. [14]	2005	Comparative genomic analysis of Clade I and Clade II MPXV	Identified nine proteins differing by at least five amino acids between Clade I and Clade II, suggesting functional roles in virulence and immune evasion	Foundational study that established early genomic differences between clades I and II
Chen et al. [21]	2005	Virulence study using West African and Central African isolates	Showed that virulence differences stem from genes located in terminal regions of the viral genome, particularly D14L, which is absent in Clade II	Provided molecular evidence for why Clade I has higher pathogenicity
O'Toole et al. [32]	2023	Evolutionary analysis of APOBEC3-type mutations in MPXV	Found sustained human-to-human transmission due to host-induced mutations, leading to Clade 1b′s enhanced fitness	Crucial study showing how Clade 1b is adapting to human hosts
Yu et al. [8]	2023	Phylogenetic study on MPXV evolution (2021–2023)	Demonstrated a rapid increase in conserved amino acid mutations, enhancing MPXV adaptation to humans	Highlights the unusual and accelerated evolution of Clade 1b
Duarte et al. [3]	2024	Comprehensive literature review on MPXV	Confirmed Clade 1b′s high mortality in children and ability to sustain human transmission	Supports epidemiological concerns about Clade 1b′s spread
Schwartz [10]	2024	Clinical study on MPXV in pregnant women	Documented high rates of miscarriage and stillbirths associated with Clade I infections	Raises concerns about the severe impact of Clade 1b on reproductive health
Masirika et al. [6]	2024	Genome sequencing and mutation profiling	Identified key mutative proteins (C9L, I4L, L6R, A17L, A25R, A28L, B21R) responsible for virulence and immune evasion	Provides crucial insights into the molecular mechanisms behind Clade 1b′s pathogenicity
Yeh et al. [33]	2022	SNP analysis of recombination events in MPXV	First study to report natural recombination in MPXV, which may be influencing Clade 1b′s evolution	Raises the possibility of MPXV evolving further through recombination
WHO	2024	Global outbreak monitoring and surveillance	Documented spread of Clade 1b to nonendemic countries (Sweden, Thailand, Rwanda, Uganda)	Supports concerns about Clade 1b as a global health threat

## Data Availability

Data sharing is not applicable to this article as no new data were created or analyzed in this study.

## Nomenclature

MPox: Monkeypox
MPXV: Monkeypox virus
DRC: Democratic Republic of Congo
CDC: Centers for disease control and prevention
WHO: World health organization
PHEIC: Public health emergency of international concern
CFR: Case fatality rate
GBMSM: Gay, bisexual, and men who have sex with men
ORF: Open reading frame
RR: Ribonucleotide reductase
APOBEC3: Apolipoprotein B mRNA editing catalytic polypeptide-like 3
SNP: Single nucleotide polymorphism
LD: Linkage disequilibrium
TR: Tandem repeat
ESM: Early stop mutation
VAVC: Vaccinia virus
PKR: Protein kinase R
MSM: Men who have sex with men
CAR: Central African Republic
EGFP-GPT: Enhanced green fluorescent protein-guanine phosphoribosyltransferase
MOPICE: Monkeypox inhibitor of complement enzymes
OPG: Orthopoxvirus gene
NCBI: National Center for Biotechnology Information
EMBL-EBI: European molecular biology laboratory-European bioinformatics institute
MMWR: Morbidity and mortality weekly report
NHS: National Health Service
RNA: Ribonucleic acid
DNA: Deoxyribonucleic acid
bp: Base pairs
CFA: Centers for forecasting and analytics
